# Pregnancy in women with Hereditary Angioedema due to C1-inhibitor deficiency: Results from the ITACA cohort study on outcome of mothers and children with *in utero* exposure to plasma-derived C1-inhibitor

**DOI:** 10.3389/fmed.2022.930403

**Published:** 2022-09-14

**Authors:** P. Triggianese, R. Senter, A. Petraroli, A. Zoli, M. Lo Pizzo, D. Bignardi, E. Di Agosta, S. Agolini, F. Arcoleo, O. Rossi, S. Modica, E. Greco, M. S. Chimenti, G. Spadaro, C. De Carolis, M. Cancian

**Affiliations:** ^1^Rheumatology, Allergology and Clinical Immunology, Department of “Medicina dei Sistemi”, University of Rome Tor Vergata, Rome, Italy; ^2^Department of Systems Medicine, University Hospital of Padua, Padua, Italy; ^3^Centro Interdipartimentale di Ricerca in Scienze Immunologiche di Base e Cliniche (CISI) dell'Università degli Studi di Napoli Federico II, Naples, Italy; ^4^Azienda Ospedaliera Universitaria Ospedali Riuniti di Ancona, Ancona, Italy; ^5^Azienda Ospedaliera Ospedali Riuniti Villa Sofia-Cervello, Palermo, Italy; ^6^Department of Medicine Integrated With the Territory, Ospedale Policlinico San Martino, IRCCS, Genoa, Italy; ^7^Department of Experimental and Clinical Medicine, University of Florence, Florence, Italy

**Keywords:** C1-inhibitor, hereditary angioedema, education, counseling, pregnancy

## Abstract

**Background:**

In women with Hereditary Angioedema (HAE) due to C1-inhibitor (C1INH) deficiency (C1INH-HAE), pregnancy counseling and treatment can be challenging. Despite the evidence of the immediate favorable outcome and safety of plasma-derived (pd)C1INH concentrate, there are no data regarding any difference among women who underwent or not pdC1INH during pregnancy or on children with *in utero* exposure to pdC1INH. The present interview study aimed at analyzing outcome of C1INH-HAE mothers and children according to pdC1INH-exposure during pregnancies.

**Methods:**

C1INH-HAE women who experienced at least 1 pregnancy were included from seven centers of the Italian Network for Hereditary and Acquired Angioedema (ITACA). The interview study retrospectively analyzed pregnancies who underwent (group 1) or not (group 2) pdC1INH. The overall goals of the study included immediate and long-term outcomes, in terms of outcomes in the time interval between pregnancy and survey.

**Results:**

A total of 168 pregnancies from 87 included women were analyzed. At term delivery (>37 gestation-week, GW) has been registered in 73.8% of cases, while spontaneous abortion (SA) occurred in 14.2% of cases with a mean GW 7 ± 2. The group 1 including pdC1INH-treated pregnancies comprised a third of the cohort (51/168, time interval 1.5 ± 10.4 yrs), while the group 2 represented 69.6% (117/168, time interval 32.8 ± 14 yrs). The same prevalence of SA occurred when comparing group 1 (11.7%) with group 2 (15.4%) with a similar GW at SA. The group 1 was older at the pregnancy time and younger at the interview than the group 2 (*P* < 0.01 for both); moreover, the group 1 showed a higher prevalence of cesarean delivery (*P* < 0.0001). The overall prevalence of obstetrical syndromes was similar between two groups: however, gestational diabetes was described only in pdC1INH-untreated pregnancies. *In utero* pdC1INH-exposed children (*n* = 45) did not show differences compared with unexposed ones (*n* = 99) in neonatal short-term outcomes.

**Conclusion:**

Through appropriate management and counseling, most of C1INH-HAE women undergo successful pregnancy and delivery. For pregnant C1INH-HAE women being treated with pdC1INH, our findings are reassuring and might lead to an improvement of both the knowledge about treatments and the experience of HAE itself.

## Introduction

Hereditary angioedema (HAE) resulting from the defect of C1 inhibitor (C1INH) is an autosomal dominant disease characterized by recurrent attacks of cutaneous and submucosal swelling in any site which are generally self-limiting within 72 h (1, 2). The defect of C1INH comprises either a deficiency (type I C1INH-HAE) or a dysfunction (type II C1INH-HAE) of the protein, allowing for a dysregulated plasma kallikrein activity within the kallikrein–kinin pathway, and thus for the overproduction of bradykinin with, in turn, the consequent activation of the bradykinin B2 receptors (1, 2). The resulting increased vascular permeability induces angioedema attacks that recognize several triggers including stress, infections, and estrogens (3, 4). In this context, women with C1INH-HAE experience a greater incidence of attacks and a stronger clinical severity with poorer quality of life than male patients (5). Women can be asymptomatic until puberty, while the exposure to increased concentrations of estrogens–both endogenous (puberty, menstrual cycle, and pregnancy) and/or exogenous (hormonal medications)—can trigger recurrent angioedema attacks (6). Although no epidemiologic studies showed a higher prevalence of reproductive failure in HAE women, previous evidence described few cases of C1INH-HAE women with abnormalities in both complement components and ovarian function, thus suggesting an intriguing interplay between kallikrein–kinin pathway and fertility (7). Moreover, as documented by data from the literature, a dysregulated complement system (CS) acts as a key factor in the pathogenesis of several obstetrical complications including early pregnancy loss, pre-eclampsia, and pre-term birth (8, 9). Therefore, in women with C1INH-HAE, pregnancy can be challenging because of the complex network among dysregulated plasma kallikrein activity, estrogens, and fertility. Nevertheless, reproductive outcome in C1INH-HAE women shows variable course, with the plasma-derived (pd)C1INH being the only specific treatment of angioedema attacks during pregnancy for both on demand and prophylaxis management (4, 5). Despite the evidence on the immediate favorable outcome as well on the safety of pdC1INH concentrate during pregnancy/labor (10–12), no data have been registered concerning differences among pregnancies who underwent or not pdC1INH or children with/without *in utero* exposure to pdC1INH.

Hence, the main aim of the present interview study was to explore outcomes of C1INH-HAE mothers and their children according to the exposure to pdC1INH during pregnancies in both therapeutic regimens: on demand versus long-term prophylaxis.

## Patients and methods

We performed a retrospective cross-sectional study via an interview regarding pregnancy data of C1INH-HAE women who had experienced at least 1 pregnancy. The study was performed during a 12-month period (2021). Seven centers of the Italian Network for Hereditary and Acquired Angioedema (ITACA) participated in the study (13). Inclusion criteria were: (a) female patients with a defined diagnosis of C1INH-HAE (1, 2), and 2) history of at least one pregnancy. Exclusion criteria consisted of pre-pubertal age of female patients, female infertility, no wish for offspring, no consent to study. The interview study thus analyzed pregnancies that underwent (group 1) or not (group 2) pdC1INH.

The overall goal of the study was to compare the immediate pregnancy outcomes in accordance with the *in-utero* exposure to pdC1INH. The primary outcomes were: delivery (at-term or pre-term), occurrence of obstetrical syndromes for mothers, birth weight and Apgar for children. In addition, secondary outcomes included the prevalence of C1INH-HAE diagnosis from both mothers and living children, as well as other concomitant diseases at the last follow up. A semi-structured survey was developed, and the main items were selected to obtain data on pregnancy outcomes and pdC1INH treatment. Both quantitative and qualitative data were collected by using the semi-structured interview that was applied by experienced researchers at each ITACA center involved in the study. Data about C1INH-HAE comprised: age at onset of symptoms and diagnosis, triggers, frequency of angioedema attacks, C1INH-HAE diagnosis before pregnancy, C1INH-HAE treatment during pregnancies. Specific questions on pregnancies included: time of the occurrence of pregnancies and mother's age at pregnancy, type of pregnancy (spontaneous, assisted reproductive techniques, e.g. *in vitro* fertilization), occurrence of spontaneous abortion [SA, defined as a spontaneous pregnancy loss before 20 gestation-week (GW)], at term delivery (> 37 GW), pre-term delivery (≤ 37 GW) (14, 15), obstetrical syndromes [including hypertensive disorders of pregnancy (HDP), eclampsia, gestational diabetes], placental abnormalities (16–18). HAE severity was defined in accordance with frequency of angioedema attacks: high disease activity was defined as ≥1 attack/4 weeks. Both long-term prophylaxis (LTP) and short-term prophylaxis (STP), as well as on demand treatment of attacks during pregnancy were registered.

We included the following newborn outcomes: birth weight (normal ≥2.5 kg, low <2.5 kg, high ≥4.5 kg), Apgar score (normal range 7–10), breastfeeding (yes/no), C1INH-HAE diagnosis, congenital abnormalities, concomitant diseases (autoimmune systemic diseases and allergy) at the time of the survey (17, 19). The Local Ethics Committees approved the study and every patient provided informed consent at each ITACA center involved in the study.

### Statistical analysis

The data were entered anonymously into a database and a descriptive statistical analysis was performed. Continuous data were expressed as mean (SD) if the distribution was normal; categorical variables were expressed as counts and percentages. Continuous variables were compared by using either the parametric unpaired *T*-test or the non-parametric Mann–Whitney U test, as appropriate. Categorical variables were compared using the Chi-squared test or Fisher's exact test, as appropriate. The *p*-values <0.05 were considered significant. All statistical analyses were performed using the GraphPad Prism version 9 (GraphPad software).

## Results

### Characteristics of study cohort

Eighty-seven female patients out of 234 followed-up in the 7 ITACA centers participating in the study fulfilled the inclusion criteria. The remaining women (*n* = 147) were excluded mainly for no consent to interview (36.7%) or no wish for offspring (34%), whereas pre-pubertal age (17.7%) and primary infertility (11.6%) represented minor cases.

A total of 168 pregnancies related to 87 women were analyzed (Table 1). All included women were type I C1INH-HAE and, in almost all the pregnancies, we registered C1INH-HAE familial history. Nearly all pregnancies were spontaneous (98.2%), with first pregnancies in a half of cases (50.6%). At term deliveries were reported in 73.8% of cases, while SA occurred in 14.3% with a mean GW 7 ± 1.9. Pre-term deliveries ( ≤ 37 GW) occurred in 12% of pregnancies. Obstetrical complications were reported in a quarter of the whole cohort (Table 1) and were mainly represented by preterm delivery (PD) (58.8%) and placental abnormalities (26.4%), whereas complicated labor by HAE acute attacks occurred in 11.7% of the pregnancies. The occurrence of other therapies during pregnancy has been documented in rare cases (14/168) and was levothyroxine supplementation for concomitant thyroiditis in half the cases and low-dose aspirin as isolated intervention for preventing obstetrical syndromes in the remaining cases.

**Table 1:** Data from pregnancies in the study sample.

	**Pregnancies** **(*N* = 168)**	**pdC1INH, YES** **(*N* = 51)**	**pdC1INH, NO** **(*N* = 117)**
Mothers' age at pregnancy, yrs (mean ± SD)	26.5 ± 7.9	29.8 ± 5.4**	26 ± 4
Mothers' age at the study, yrs (mean ± SD)	48.5 ± 16.9	41.3 ± 7.7**	55.2 ± 14.2
Mothers' C1INH-HAE diagnosis after pregnancy (*N*/%)	73/43.5	3/5.8****	70/59.8
First pregnancy (*N*/%)	85/50.6	24/47	61/52.1
Cesarean delivery (*N*/%)	43/29.9	25/49****	18/15.4
At term delivery, >37 GW (*N*/%)	124/73.8	37/72.5	87/74.4
GW at delivery (mean ± SD)	39.2 ± 1.7	38.9 ± 1.6	38 ± 1.3
SA (*N*/%)	24/14.3	6/11.7	18/15.4
GW at SA (mean ± SD)	7 ± 1.9	8 ± 1.7	9 ± 1.3
**Obstetrical complications**			
Preterm, ≤ 37 GW (*N*/%)	20/11.9	8/17.8	12/12.2
Gestational Diabetes (*N*/%)	5/3.5	–	5/5
HDP (N/%)	3/2	3/6.7	–
Placental abnormalities (*N*/%)	9/6.3	4/8.9	5/5
HAE acute attacks ^§^(*N*/%)	4/2.7	1/2.3	3/3

C1INH, C1 inhibitor; HAE, hereditary angioedema; pdC1INH, plasma derived C1 inhibitor; GW, gestational week; SA, spontaneous abortion; HDP, hypertensive disorders of pregnancy; § at the delivery; **P < 0.01, ****P < 0.0001.

### Pregnancy outcome according to pdC1INH treatment

Approximately one third of the pregnancies (*n* = 51) underwent treatment with pdC1INH (group 1), while 117 (69.6%) were not treated with pdC1INH (group 2). In group 1 pdC1INH was administered in accordance with the disease severity: in pregnancies with a high disease severity (*n* = 7, 13.7%) it was administered as LTP (1000 UI pdC1INH, every 4 days) plus on-demand, whereas in the remaining cases it was used exclusively on demand (86.3%, 1500 UI pdC1INH). The pdC1INH was used as STP in all cases of elective cesarean deliveries in group 1. In order to explore the potential effect of the total amount of pdC1INH used during pregnancy, we additionally analyzed the few pregnancies on LTP and no significant difference in terms of prevalence of obstetrical syndromes occurred between LTP-pregnancies and on demand C1INH-pregnancies. The time interval between pregnancy and survey in the group 1 was significantly lower than in the group 2 (1.5 ± 10.4 yrs vs. 32.8 ± 14 yrs, p 0.01).

The prevalence of SA was similar in group 1 and 2 and with no differences in GW at the SA (Table 1). There were neither any significant differences regarding at term pregnancies between the two groups (Table 1). However, the mothers' mean age at pregnancy was lower in group 2 than in group 1 (*P* < 0.01), while patients were younger at the time of the interview in group 1 (*P* < 0.01). Interestingly, group 1 showed a significantly lower prevalence of HAE diagnosis after pregnancy than group 2 (*P* < 0.0001), as well as a higher prevalence of cesarean delivery (*P* < 0.0001). The overall prevalence of obstetrical syndromes was similar in the two groups, whereas gestational diabetes was only described in pdC1INH-untreated pregnancies (Table 1).

### Children outcomes according to the *in utero* pdC1INH exposure

Data from 67 living children from all pregnancies were recorded (Table 2). A normal birth weight and Apgar score were documented in both *in utero* pdC1INH exposed and unexposed newborns. Also, the prevalence of breastfeeding was similar in both groups of infants (Table 2). At the time of the survey the age of pdC1INH exposed subjects was significantly lower than the age of unexposed ones (*P* < 0.01). Accordingly, the C1INH-HAE diagnosis has not been carried out in a higher proportion in pdC1INH exposed subjects than in unexposed ones (*P* < 0.001) while a defined C1INH-HAE diagnosis was documented in a higher prevalence among unexposed subjects (*P* < 0.01).

**Table 2:** Data from newborns in accordance with pdC1INH treatment.

	**pdC1INH, YES** **(*N* = 45)**	**pdC1INH, NO** **(*N* = 99)**
Normal birth weight (*N*/%)	37/82.2	87/87.8
Breastfeeding (*N*/%)	28/62.2	79/79.8
C1INH-HAE affected (*N*/%)	14/31.2 **	59/59.6
C1INH-HAE not affected (*N*/%)	20/44.4	33/33.4
C1INH-HAE diagnosis not carried out (*N*/%)	11/24.4 ***	7/7
Congenital abnormalities (*N*/%)	0/0	2/2
Systemic autoimmune diseases (*N*/%)	0/0	2/2
Allergy (*N*/%)	2/4.4	10/10.1
Other (*N*/%)	1/2.2	7/7
Current age, yrs (mean ± SD)	11.5 ± 10.4 **	32.8 ± 14

C1INH, C1 inhibitor; HAE, hereditary angioedema; pdC1INH, plasma derived C1 inhibitor.

^**^P 0.01 and ^***^P 0.001 with the respect to pdC1INH NO group.

## Discussion

### Pregnancy outcomes in C1INH-HAE women

Pregnancy outcome in C1INH-HAE women is still a challenge considering the effects that hormones and pregnancy itself have on complement and kallikrein–kinin pathway activation, that may worsen disease activity (20, 21). Moreover, considering the role of immune-mediated pathways in women reproduction, the dysregulated kallikrein–kinin pathway in C1INH-HAE women might potentially predispose to a worse pregnancy outcome (8).

The disease inheritance as well as the impact of unpredictable recurrent acute angioedema attacks might influence the family planning (22). Therefore, nearly a quarter of the C1INH-HAE women followed-up at the involved ITACA centers was not eligible for the interview study because of no wish for offspring. However, they represented a relatively small group of the C1INH-HAE women, probably because the presence of family history of HAE in almost all the registered pregnancies led to adequate self-awareness of the disease.

In accordance with data from the literature, reproductive failure in terms of infertility and SA was not prevalent in our sample of C1INH-HAE pregnancies (5, 23). We documented that nearly all pregnancies were spontaneous and three-quarters of them experienced at term delivery. The occurrence of PD and SA in our cohort recognized a prevalence similar to that of non-C1INH-HAE women of child-bearing-age and this result may be associated with adequate therapeutic management (5, 6, 24). According to the data collected, all pregnancies with obstetric complications experienced such comorbidities, including PDH and metabolic abnormalities, only during the weeks of gestation and represented a very small percentage (10%). A subanalysis of pregnant women who suffered from hypertension could provide interesting insights on the effects of comorbidities on pregnancy outcome: however, few cases have been documented in the present study. These issues could be adequately addressed by further investigations of a larger cohort with a prospective study design.

### The use of pdC1INH during pregnancy

One-third of all pregnancies in our population underwent pdC1INH for both treatment of acute HAE attacks and for prophylaxis, regardless of a specific gestational trimester. It might be surprising that two-thirds of all pregnancies had not received pdC1INH; however, more than half of them were classified as affected by C1INH-HAE after pregnancy and were therefore managed differently (5). Nevertheless, group 2 C1INH-HAE diagnosed pregnancies did not use pdC1INH due to low disease severity, suggesting that although pregnancy has been considered as a triggering factor for HAE attacks, it may have a good effect on HAE disease activity (5). As previously described, it is rare for clinical manifestations of C1INH-HAE to present for the first time during pregnancy; however, C1INH levels decrease during pregnancy in relation to increased plasma volume, and transient low levels of C1INH have been described in pregnant women without C1INH-HAE, making its diagnosis difficult during pregnancy (5). In our cohort, few group 1 pregnancies received pdC1INH without a definite diagnosis of C1INH-HAE because they had experienced angioedema attacks for the first time during pregnancy. However, the presence of a family history of HAE represented a high index of suspicion in all these cases, which allowed appropriate treatment with pdC1INH of the angioedema attacks. After pregnancy, all of these women received a certified diagnosis of C1INH-HAE. As documented, our results support the use of pdC1INH to achieve a favorable outcome in women with C1INH-HAE who experience worsening disease severity during gestation (23, 25–28).

Additionally, international guidelines support the use of pdC1INH as a long-term prophylaxis also in pregnant C1INH-HAE women with histories of miscarriage and/or high-risk pregnancies with a suggested dosage being the same as in nonpregnant patients despite the pregnancy weight gain (5). Consistent with our results, there were no differences in SA or PD rates between pdC1INH-treated and untreated pregnancies and no differences in delivery week in at term pregnancies. The reasons for PD have not been further defined as they were not related to acute HAE attacks or documented cardiovascular and/or metabolic disorders. Metabolic disorders presented a different prevalence between pdC1INH-treated and untreated pregnancies, showing a higher frequency in the latter group probably due to the use of steroids to treat angioedema attacks in group 2 women without a definite diagnosis of C1INH-HAE during pregnancy.

Furthermore, there was a significant difference between the groups with respect to the mean age of the women at the time of the study, since those who had received pdC1INH in pregnancy were younger than those who had not received pdC1INH in pregnancy. This difference suggests that women with C1INH-HAE who had a more recent pregnancy were more willing to treat angioedema attacks or had more medications available than women who had had a pregnancy in the past. In addition, our findings indirectly highlighted a gradual reduction in diagnostic delay over time in C1INH-HAE patients, as women in group 1 were younger and showed a higher rate of C1INH-HAE diagnosis before pregnancy compared to women in group 2.

### Delivery in pregnant C1INH-HAE women

Even though cute attacks of HAE complicated few deliveries, management of HAE during labor requires special consideration because it may be exacerbated (6, 25). As expected, since all pregnancies with cesarean delivery and pdC1INH-STP were treated with pdC1INH (at least as STP during delivery), elective cesarean delivery with pdC1INH-STP was more common for pdC1INH-treated than untreated pregnancies. Cesarean delivery is not recommended in women with C1INH-HAE, so its higher prevalence in pregnancies treated with pdC1INH and therefore in younger women with HAE could reflect an increase in the indication of cesarean sections over time (22, 29–31).

As has been published, in women with C1INH-HAE, vaginal deliveries are preferred over cesarean sections, and in this setting, epidural anesthesia is preferred over general anesthesia to reduce the risk of acute attacks of angioedema (6). Nevertheless, labor and delivery only rarely induce an attack, which could occur either during labor or within 48 h of delivery (32). In our view, elective cesarean section, although not absolutely advisable, could allow for adequate and targeted management of delivery in selected women with C1INH-HAE, mainly those with high severity of the disease and/or on LTP, to avoid emergent deliveries that could be complicated by difficult-to-treat angioedema attacks. Furthermore, pdC1INH should always be available in the delivery room, also in vaginal deliveries (5, 6).

### Children outcomes and intrauterine pdC1INH exposure

Live birth data documented normal birth weight and Apgar score in neonates exposed and unexposed to pdC1INH *in utero*. These data directly support the immediate safety of pdC1INH during pregnancy as reported by evidence from the literature (23, 26). At the time of the survey study, the occurrence of a definite C1INH-HAE diagnosis had occurred at a lower rate among pdC1INH-exposed than unexposed neonates, resulting in a lower prevalence of C1INH-HAE in the first group of babies (32–34). The reason was related to the younger age of the C1INH-HAE women who underwent pdC1INH during pregnancy and, therefore, the younger age of their children. In C1INH-HAE, prenatal diagnosis in established pregnancy is only rarely requested and can only be performed if the disease-causing mutation of the affected parent is known (5). Nevertheless, C1INH-HAE diagnosis in neonates and infants can be performed by using biochemical tests that may be inconclusive in very young children (<12 months). Genetic testing is thus a safer and more direct tool to determine whether a child has inherited the disease and in newborns it can be performed on umbilical cord or peripheral blood (5). In addition, biochemical and genetic testing of asymptomatic children with affected parent should be performed because presymptomatic C1INH-HAE children are at risk of unexpected attacks and the early diagnosis can help to ensure the adequate treatment (28). However, live birth data among neonates described that the occurrence of childhood deficiencies was uncommon and independent of intrauterine exposure to pdC1INH.

C1INH-HAE could produce autoimmunity due to the consumption of early components of the classical complement pathway, as in patients with genetic C1 or C2 deficiencies (35). It can be hypothesized that pdC1INH replacement therapy may have a modulatory impact on autoimmune diseases in C1INH-HAE (occurrence and/or severity) by increasing C1-INH, C4, and/or C2 as suggested in some studies (36). Similarly, the role of other complement components such as C3a and C5a as potential effectors in type 1 hypersensitivity reactions, as well as crosstalk between mast cells and complement suggest that complement activation may also synergize with classical IgE responses, possibly affecting allergic disorders (37, 38). Consequently, we analyzed the prevalence of autoimmune and allergic diseases in the two groups of children at the last follow-up (interview-time), and there were no differences. However, the different ages and the restricted population represent relevant limitations for these results that should certainly be confirmed by future clinical investigations. Further analysis on the occurrence of allergic and/or autoimmune diseases in a larger HAE sample and/or in the general population should be addressed in prospective studies that stratify children according to the co-diagnosis of C1INH- HAE.

### Limitations and strengths

The main limitation of our results is represented by the retrospective design of the study that included women whose pregnancy had occurred more than 30 years earlier. The long-time interval in some cases could have given rise to a forgetting bias and consequently a lack of data and objective information on the use of pdC1INH, concomitant treatments and/or obstetric complications. For instance, the total amount of pdC1INH used during pregnancies was not available from our collected data due to bias related to the retrospective design. However, the exact amount of pdC1INH administered in each pregnancy would certainly have improved the quality of the results: therefore, further investigations with a prospective design should focus on the specific accumulated amount of received pdC1INH and its possible correlation with the outcomes.

Nevertheless, the retrospective design allowed us to focus on multiple data at the same time and long-term patient history, which could provide information on the course and burden of the disease, outcomes and therapeutic management over the years. Anyway, the main strength of the present study is represented by its multicenter design, which made it possible to obtain a representative and relevant sample of patients with such a rare disease.

### Conclusions

Reproductive planning is a persistent concern for women with inherited and rare diseases (29–31). As known, mechanisms of reproductive failure involve immune-mediated pathways including dyregulated complement and kallikrein–kinin pathway, mainly locally, at the site of implant, also in women without C1INH-HAE (8, 39, 40). Accordingly, the ultimate goal for C1INH-HAE management, particularly during pregnancy, is to achieve disease remission and no attacks and thus to use appropriate treatments making the complete control of HAE a realistic possibility for patients (41–43). For pregnant C1INH-HAE women being treated with pdC1INH, our findings are reassuring and might lead to an improvement of both the knowledge about treatments and the experience of HAE itself.

## Data availability statement

The raw data supporting the conclusions of this article will be made available by the authors, without undue reservation.

## Ethics statement

The study involving human participants was reviewed and approved by each local committee at the involved ITACA Centers. Written informed consent to participate in this study was provided by the participants' legal guardian/next of kin.

## Author contributions

PT, GS, RS, and MCa contributed to the conceptualization and scope. PT, RS, and MCa read and approved the final version. All authors collected and analyzed data, contributed to the article, and approved the submitted version.

## Conflict of interest

Author MCa received speaker/consultancy fees from BioCryst, CSL Behring, Pharming, and Takeda. Authors PT and MCa received speaker/consultancy fees from CSL Behring. Author RS received grants from CSL Behring and Takeda. The remaining authors declare that the research was conducted in the absence of any commercial or financial relationships that could be construed as a potential conflict of interest.

## Publisher's note

All claims expressed in this article are solely those of the authors and do not necessarily represent those of their affiliated organizations, or those of the publisher, the editors and the reviewers. Any product that may be evaluated in this article, or claim that may be made by its manufacturer, is not guaranteed or endorsed by the publisher.

## References

[1] Zanichelli A Arcoleo F Barca MP Borrelli P Bova M Cancian M. A nationwide survey of hereditary angioedema due to C1 inhibitor deficiency in Italy. Orphanet J Rare Dis. (2015) 10:11. 10.1186/s13023-015-0233-x 25758562 PMC4333895

[2] Zuraw BL. Hereditary angioedema. N Engl J Med. (2008) 359:1027–36. 10.1056/NEJMcp0803977 18768946

[3] Nussberger J Cugno M Amstutz C Cicardi M Pellacani A Agostoni A. Plasma bradykinin in angio-oedema. Lancet. (1998) 351:1693–7. 10.1016/S0140-6736(97)09137-X 9734886

[4] Maurer M Magerl M Ansotegui I Aygören-Pürsün E Betschel S Bork K. The international WAO/EAACI guideline for the management of hereditary angioedema-the 2017 revision and update. Allergy. (2018) 73:1575–96. 10.1111/all.13384 29318628

[5] Caballero T Farkas H Bouillet L Bowen T Gompel A Fagerberg C. International consensus and practical guidelines on the gynecologic and obstetric management of female patients with hereditary angioedema caused by C1 inhibitor deficiency. J Allergy Clin Immunol. (2012) 129:308–20. 10.1016/j.jaci.2011.11.025 22197274

[6] Yakaboski E Motazedi T Banerji A. Hereditary angioedema: special considerations in women. Allergy Asthma Proc. (2020) 41:S47–50. 10.2500/aap.2020.41.200077 33109327

[7] Perricone R De Carolis C Giacomello F Giacomelli R De Sanctis G Fontana L. Impaired human ovarian follicular fluid complement function in hereditary angioedema. Scand J Immunol. (2000) 51:104–8. 10.1046/j.1365-3083.2000.00652.x 10632984

[8] Triggianese P Perricone C Chimenti MS De Carolis C Perricone R. Innate immune system at the maternal-fetal interface: mechanisms of disease and targets of therapy in pregnancy syndromes. Am J Reprod Immunol. (2016) 76:245–57. 10.1111/aji.12509 27108670

[9] Cavalli S Lonati PA Gerosa M Caporali R Cimaz R Chighizola CB. Beyond systemic lupus erythematosus and anti-phospholipid syndrome: the relevance of complement from pathogenesis to pregnancy outcome in other systemic rheumatologic diseases. Front Pharmacol. (2022) 13:841785. 10.3389/fphar.2022.841785 35242041 PMC8886148

[10] Banerji A Riedl M. Managing the female patient with hereditary angioedema. Women's Health. (2016) 12:351–61. 10.2217/whe.16.6 26978558 PMC5384520

[11] Brooks JP Radojicic C Riedl MA Newcomer SD Banerji A Hsu FI. Experience with intravenous plasma-derived C1-inhibitor in pregnant women with hereditary angioedema: a systematic literature review. J Allergy Clin Immunol Pract. (2020) 8:1875–80.e3. 10.1016/j.jaip.2020.03.009 32251736

[12] Baker J Sheffer A Christensen J Hurewitz D Lazar R Kalfus I. CinryzeTM replacement therapy in hereditary angioedema and pregnancy. J Allergy Clin Immunol Pract. (2009) 123:S106. 10.1016/j.jaci.2008.12.385

[13] Cancian M. Italian network for C1-INH-HAE (ITACA). Diagnostic and therapeutic management of hereditary angioedema due to C1-inhibitor deficiency: the Italian experience. Curr Opin Allergy Clin Immunol. (2015) 15:383–91. 10.1097/ACI.0000000000000186 26106828

[14] Practice Committee of the American Society for Reproductive Medicine. Evaluation and treatment of recurrent pregnancy loss: a committee opinnion. Fertility Sterility. (2012) 98:1103–11. 10.1016/j.fertnstert.2012.06.048 22835448

[15] Rai R. Regan L. Recurrent miscarriage. Lancet. (2006) 368:601–11. 10.1016/S0140-6736(06)69204-0 16905025

[16] Magee LA Helewa M Rey E. Diagnosis, evaluation, and management of the hypertensive disorders of pregnancy. J Obstet Gynaecol Can. (2008) 30:S1–2. 10.1016/S1701-2163(16)32870-5 18817592

[17] Triggianese P Lattavo G Chimenti MS Conigliaro P Perricone R Perricone C. Reproductive outcomes 20 years after the intravenous immunoglobulin treatment in women with recurrent pregnancy losses. Am J Reprod Immunol. (2020) 83:e13224. 10.1111/aji.13224 31971295

[18] Athukorala C Rumbold AR Willson KJ Crowther CA. The risk of adverse pregnancy outcomes in women who are overweight or obese. BMC Pregnancy Childbirth. (2010) 10:56. 10.1186/1471-2393-10-56 20849609 PMC2949787

[19] Zong X Wang H Yang L Guo Y Zhao M Magnussen CG Xi B. Maternal pre-pregnancy body mass index categories and infant birth outcomes: a population-based study of 9 million mother-infant pairs. Front Nutr. (2022) 9:789833. 10.3389/fnut.2022.789833 35252291 PMC8891137

[20] Bouillet L. Hereditary angioedema in women. Allergy Asthma Clin Immunol. (2010) 6:17. 10.1186/1710-1492-6-17 20667120 PMC2918592

[21] Visy B Füst G Varga L Szendei G Takács E Karádi I. Sex hormones in hereditary angioneurotic oedema. Clin Endocrinol. (2004) 60:508–15. 10.1111/j.1365-2265.2004.02009.x 15049967

[22] González-Quevedo T Larco JI Marcos C Guilarte M Baeza ML Cimbollek S. Management of pregnancy and delivery in patients with hereditary angioedema due to C1 inhibitor deficiency. J Investig Allergol Clin Immunol. (2016) 26:161–7 10.18176/jiaci.0037 27326983

[23] Czaller I Visy B Csuka D Füst G Tóth F Farkas H. The natural history of hereditary angioedema and the impact of treatment with human C1-inhibitor concentrate during pregnancy: a long-term survey. Eur J Obstet Gynecol Reprod Biol. (2010) 152:44–9. 10.1016/j.ejogrb.2010.05.008 20541309

[24] Nybo Andersen AM Wohlfahrt J Christens P Olsen J Melbye M. Maternal age and fetal loss: population based register linkage study. BMJ. (2000) 320:1708–12. 10.1136/bmj.320.7251.1708 10864550 PMC27416

[25] Geng B Riedl MA. HAE update: special considerations in the female patient with hereditary angioedema. Allergy Asthma Proc. (2013) 34:13–22. 10.2500/aap.2013.34.3635 23406930

[26] Farkas H Csuka D Toth F Koszegi L Varga L. Successful pregnancy outcome after treatment with C1-inhibitor concentrate in a patient with hereditary angioedema and a history of four miscarriages. Eur J Obstet Gynecol Reprod Biol. (2012) 165:366–7. 10.1016/j.ejogrb.2012.07.010 22884590

[27] Hakl R Kuklínek P Krčmová I Králíčková P Freiberger T Janku P. Treatment of hereditary angioedema attacks with icatibant and recombinant C1 inhibitor during pregnancy. J Clin Immunol. (2018) 38:810–5. 10.1007/s10875-018-0553-4 30280305

[28] Savarese L Bova M De Falco R Guarino MD De Luca Picione R Petraroli A. Emotional processes and stress in children affected by hereditary angioedema with C1-inhibitor deficiency: a multicenter, prospective study. Orphanet J Rare Dis. (2018) 13:115. 10.1186/s13023-018-0871-x 30005674 PMC6043996

[29] Lazzaroni MG Crisafulli F Moschetti L Semeraro P Cunha AR Neto A. Reproductive issues and pregnancy implications in systemic sclerosis. Clin Rev Allergy Immunol. (2022). 10.1007/s12016-021-08910-0 35040084

[30] Wilson RD. The real maternal risks in a pregnancy: a structured review to enhance maternal understanding and education. J Obstet Gynaecol Can. (2020) 42:1364–78.e7. 10.1016/j.jogc.2019.12.005 32712227

[31] Banerji A Li Y Busse P Riedl MA Holtzman NS Li HH. Hereditary angioedema from the patient's perspective: A follow-up patient survey. Allergy Asthma Proc. (2018) 39:212–23. 10.2500/aap.2018.39.4123 29669666 PMC5911511

[32] Maurer M Magerl M Betschel S Aberer W Ansotegui IJ Aygören-Pürsün E. The international WAO/EAACI guideline for the management of hereditary angioedema - The 2021 revision and update. Allergy. (2022) 77:1961–90. 10.1111/all.15214 35006617

[33] Farkas H Harmat G Füst G Varga L Visy B. Clinical management of hereditary angio-oedema in children. Pediatr Allergy Immunol. (2002) 13:153–61. 10.1034/j.1399-3038.2002.01014.x 12144636

[34] Cancian M Perego F Senter R Arcoleo F De Pasquale T Zoli A. Pediatric angioedema: essential features and preliminary results from the hereditary angioedema global registry in Italy. Pediatr Allergy Immunol. (2020) 31 Suppl 24:22–4. 10.1111/pai.13170 32017221

[35] Triggianese P Chimenti MS Toubi E Ballanti E Guarino MD Perricone C. The autoimmune side of hereditary angioedema: insights on the pathogenesis. Autoimmun Rev. (2015) 14:665–9. 10.1016/j.autrev.2015.03.006 25827463

[36] Farkas H Levy D Supina D Berger M Prusty S Fridman M. Hereditary angioedema C1-esterase inhibitor replacement therapy and coexisting autoimmune disorders: findings from a claims database. Allergy Asthma Clin Immunol. (2020) 16:42. 10.1186/s13223-020-00439-9 32514273 PMC7254637

[37] Gerard NP Gerard C. Complement in allergy and asthma. Curr Opin Immunol. (2002) 14:705–8. 10.1016/S0952-7915(02)00410-7 12413519

[38] Elieh Ali Komi D Shafaghat F Kovanen PT Meri S. Mast cells and complement system: Ancient interactions between components of innate immunity. Allergy. (2020) 75:2818–28. 10.1111/all.14413 32446274

[39] Triggianese P Perricone C Conigliaro P Chimenti MS Perricone R De Carolis C. Peripheral blood natural killer cells and mild thyroid abnormalities in women with reproductive failure. Int J Immunopathol Pharmacol. (2016) 29:65–75. 10.1177/0394632015615130 26657164 PMC5806729

[40] Valdés G Acuña S Munizaga A Soto GX Figueroa CD. Utero-placental cellular and nuclear expression of bradykinin B2 receptors in normal and preeclamptic pregnancies. Pregnancy Hypertens. (2016) 6:30–7. 10.1016/j.preghy.2016.01.003 26955769

[41] Maurer M Aygören-Pürsün E Banerji A Bernstein JA Balle Boysen H Busse PJ. Consensus on treatment goals in hereditary angioedema: A global Delphi initiative. J Allergy Clin Immunol. (2021) 148:1526–32. 10.1016/j.jaci.2021.05.016 34048855

[42] Savarese L Bova M Maiello A Petraroli A Mormile I Cancian M. Psychological processes in the experience of hereditary angioedema in adult patients: an observational study. Orphanet J Rare Dis. (2021) 16:23. 10.1186/s13023-020-01643-x 33422102 PMC7796642

[43] Savarese L Freda MF De Luca Picione R Dolce P De Falco R Alessio M. The experience of living with a chronic disease in pediatrics from the mothers' narratives: the clinical interview on parental sense of grip on the disease. Health Psychol Open. (2020) 7:2055102920971496. 10.1177/2055102920971496 33343914 PMC7727074

